# Report of Committee on Insanity

**Published:** 1869-09

**Authors:** 


CHICAGO MEDICAL EXAMINER.

N. S. DAVIS, M.D., EDITOR.

VOL. X. SEPTEMBER, 1869. NO. 9.

### Original Contributions.

ARTICLE XXXIII.

### REPORT OF COMMITTEE ON INSANITY.

(Read before the Illinois State Medical Society, May, 1869.)

TO THE ILLINOIS STATE MEDICAL SOCIETY:

Your Committee on Insanity make the following report:

The resolution creating the Committee charges it with the duty of inquiring, "What legislation, if any, is necessary to secure the rights and comforts of the insane previous to their admission to hospital care and treatment?" The subject, therefore, suggests a review of the laws in force in regard to the admission or commitment of insane persons to hospitals, and the Committee limit their report to medico-legal inquiries.

The statutes of Illinois are believed to be peculiar to this State, having no precedents in the country, outside its boundaries. They demand, as a prerequisite to receiving hospital care and treatment, that every possible form and case of insanity shall have a jury trial, the insane person being compelled to appear and remain present in open court during the trial, in order to determine the fact of the existence of insanity. The questions now arise: Are the provisions of this peculiar statute necessary in order to protect persons from wrongful imprisonment? Do they secure the rights and comforts of the insane? Or is it a legislation for exceptional cases? Is the law humane in its bearings toward the insane? And if not, what legislation is desirable, as viewed from medico-legal standpoint by medical men?

That eminent jurist, Chief Justice Shaw, of the Supreme Judicial Court of Massachusetts, said: "The right to restrain an insane person of his liberty is found in that '*great law of humanity*,' which makes it necessary to confine those whose going at large would be dangerous to themselves or others. The necessity which creates the law creates the limitations of the law. If there is no right to exercise restraint for a fortnight, there is no right to exercise it for an hour; and if a man may be restrained in his own house, he may be restrained in an asylum. Besides, it is a principle in law, that an insane man has no will of his own. The question must then arise in each particular case, whether a person's own safety, or that of others, requires that he shall be restrained for a time, and whether restraint *is necessary for his restoration, or will be conducive thereto.* The restraint can continue as long as the necessity continues. This is the limitation, and the proper limitation."

Wharton and Stillè, in their Treatise on Medical Jurisprudence, say that: "There are necessarily cases of insanity, *when the health of the patient alone* requires confinement in an asylum, though there be no danger to himself or others. The law in such cases undoubtedly is, that confinement is justifiable if either the safety of the patient or others require it, or if it is necessary for his restoration to health."

Insane persons are none the less invalids than would be the case if the disease were located elsewhere than in the brain. Hospitals for insane persons are not prisons, designed for criminals, but places for the proper care and treatment of the sick. They are fitted and furnished so as to afford such safeguards, comforts, and appliances as are demanded for the best treatment of such invalids as may need to be committed to them, and who, on account of the character of the disease, cannot be as well cared for and treated at their homes. They are humane institutions, and their inmates are patients.

In other States than Illinois, what has been so aptly styled by Chief Justice Shaw as the "great law of humanity" is still recognized, in mainly intrusting the removal of insane persons from their homes, for hospital care and treatment, to the friends of the family (as counseled and advised by one or more physicians), who, more than others, are immediately interested in the welfare of the patient. But the law as it now stands in Illinois, with its cold formalities and damaging delays, interposes between the physician and his patient, between the husband and his sick wife, between the parent and his sick child, the brother and his invalid sister, and declares that in no case can an insane person receive hospital treatment within the bounds of the State, either in public or private institutions, either at public or private expense, without a public jury trial as to the fact of insanity.

In defence of the rigorous legal process now demanded, it is alleged that corrupt or incompetent physicians may be found, who, regardless or ignorant of their calling, *may* give fraudulent certificates of insanity; and that bribes *may* make the medical officers of hospitals for the insane accomplices in the crime of restraining sane persons under the guise of insanity.

Your Committee believe that the great crime of giving fraudulent certificates of insanity has never existed within the borders of the State; that false imprisonment, under the fraudulent plea of insanity, has not been substantiated in any one well authenticated case; and that the implied accusation of venality on the part of medical men is unjust. We are willing to admit that there may have been errors in diagnosis, though even this, so far as known, has not been substantiated. Patients have been sometimes adjudged insane, and so certified by medical examiners, and, when admitted to hospital, are found to have recovered, pending delays in correspondence and removal, prior to admission. But admitting that errors are liable to occur, and that bribes may procure false imprisonment, all experience and observation, throughout the whole country, go to show that the evil, if it really exists outside of morbid imaginations, must be only in the smallest possible proportions. Legislation should be for the greatest good of the overwhelming majorities; and if most rarely an exceptional error or crime may be committed, the same may be said of the opinions and acts of men in all professions, and of the various opinions of medical men in regard to other patients than those who are insane, and in regard to which the law does not by special enactment presume to interfere. False imprisonment is every day resorted to in our large commercial cities by the officers of the law themselves, upon mere suspicion of crime; and false imprisonment may occur under the present law upon the plea of insanity, where the most evenly balanced cases are submitted to a jury of unskilled men who are believed to be not the most competent men to decide any purely medical question, and especially to determine a closely contested case of insanity. In all such cases, where the patient is undemonstrative in conduct, and where the morbid mental manifestations are obscure, the jury are wholly dependent upon medical opinions, which might much more profitably go directly to the court. Unskilled juries are not allowed to be judges of questions in law science, neither are they competent, nor should they be allowed to decide questions in medical science.

It is believed by those who have had largest opportunities for observation, that in ninety-nine cases out of every hundred that go to our hospitals, probably not more than two are of so obscure a type as to cause the slightest doubt in the mind of any physician; and surely such demonstrative cases need no judicial court investigation, and no jury trial as to the fact of insanity; and in the few doubtful, obscure, or evenly-balanced cases, the jury being wholly incompetent to decide, it follows that the jury, in regard to these purely scientific medical questions, is more expensive, and more ornamental than useful.

But there are a few exceptional cases which occupy that debatable ground on one or the other side of the diffused line of demarkation between possible sanity and undoubted insanity; and these may best be determined by a special commission of medical men, or possibly by a mixed commission, as proposed by Dr. Ray, composed of both medical and legal men; one or more physicians, "in order that the medical inquiry should take the proper course, and a lawyer, that the legal proceedings should be correct, and the testimony should have legal correctness." It is a hardship and an inhumanity to subject the ninety-nine in every one hundred cases to the delays, expenses, exposures, and shock to the feelings of a jury trial in order to avoid a possible injustice to one single exceptional case which may be with ease specially provided for.

Your Committee have arrived at the opinion, that the laws in force in Illinois, in regard to the commitment of the insane for hospital treatment, are, in their application to a great majority of cases, positively damaging in their requirements and tendencies.

There are, for instance, certain cases of puerperal mania in which the patient has been subjected to the shock of parturition, drainage by hemorrhage, drainage by the lochial discharge, drainage by lactation, and still further depressed in force by abstinence from food, and loss of sleep. The case is one of excitement without force, with tendencies to fatal exhaustion. The chairman of your Committee has witnessed the trial, and I had almost said the execution, of one such case of puerperal mania, in which the excitement was terribly augmented by the presence of a gaping crowd. A lady of culture and refinement, from being comparatively quiet and manageable, gave way to loud vociferations, and finally to maniacal ravings, and the liberal use of the most obscene language, which is somewhat characteristic of these cases. The trial was as public as the most ardent admirers of the law could wish. Two men held the patient for two long hours, in the court room, awaiting the assembling of court, and the empanneling of a jury. The jurymen were selected, with one exception, from the crowd of seeming vagrants, who habitually hang about certain temples of justice in large cities. The court, in obedience to strict forms, goes through the fearful mockery of announcing to the lady on trial, and whose mind at the moment was in chaotic fury, "Madam, you are charged with being insane." "Are you ready for trial?" "Do you desire counsel?" The "professionals" in the jury-box are then supposed to listen to testimony in regard to the lady on trial, charged with having disease of the brain, and consequent morbid mental manifestations; and after having gravely asked the medical witness, whether the lady was "free from infectious disease and vermin," and whether there had "been any family quarrels of a domestic nature," and one or two other equally momentous questions, they retire for consultation. This case represents a class, and your Committee need scarcely say that such patients have no surplus strength to squander in court house exposures, and that such exhibitions are wholly unnecessary; that they are not demanded by the advancing science or civilization of the age; that they tend to jeopard the recovery of the patient, and sometimes life itself; and that they are revolting to the cultivated and humane instincts of a Christianized people.

There is another class of insane persons, some of them young females, just passing into the period of womanhood, some of them suicidal, in which the delusions are obscure, and the intellectual impairment is overshadowed by a perverted condition of the whole moral character of the individual. These timid persons need the protection and treatment which a well-conducted hospital alone can give; but to compel them to appear in open court as the subjects of inquisition, where their diseases and infirmities shall be recorded and published, and where also in connection the infirmities and infelicities of the family are often dragged to public gaze, is shocking to the feelings of the suffering patient and the family, and an unnecessary, even wanton exposure of matters which the public have no claim to know. In nearly or quite all such cases the law is, in effect, prohibitory of hospital treatment, so odious are its requirements; and the effect is to substitute home imprisonment, or at best home custody merely, for proper curative treatment.

There are also aged insane persons, and others who are feeble, some of them in whom fatal exhaustion is imminent, and where an overland journey of fifteen or twenty miles, perhaps in open wagons, and inclement seasons, to attend inquisitions, would damage the patient and jeopard recovery.

Your Committee believe that these feeble patients should not be subjected to needless fatigue, exposure, or delays, but should find needed hospital treatment through a shorter and more humane process than the law now provides. In some instances it is well known that several weeks or even months may elapse during which no court may be in session within the county where patients reside, and meantime the patient may remain in jail, associated with criminals awaiting trial, and be subjected to damaging delays, as well as damaging associations.

Your Committee are of the opinion, which accords also with all experience and observation, that, *as a rule*, insanity is only curable in its earliest stages, but that if skilful treatment be early applied, a very large majority of insane patients will recover; and that treatment in some well-managed hospital, while the case is still recent, affords the best available means for the recovery of the insane; that while every reasonable safeguard against false imprisonment should be given, laws should not be so restrictive as to result in rendering of no avail, even to the most quiet, unoffending insane person, the blessings of prompt curative treatment while the disease is amenable to treatment, and before confirmed lifelong insanity results. Your Committee believe that the laws in force put many hindrances in the way to the prompt use of those instrumentalities which are most efficient in promoting the comfort and recovery of the insane, and that its legitimate results are to leave many cases to become chronic and incurable, who should be restored to health, and that these patients, after having fruitlessly worn the families to whom they belong, and wasted their substance in vain efforts for relief, are finally thrown upon the State for a lifelong support.

As corroborative of our own views and opinions, the Committee think it not improper to introduce in this report the written opinions of several distinguished medical gentlemen who have had a large and varied experience in the treatment of the insane, extending over a period of a-quarter of a century, in which the study of the welfare of the insane has been the work of their whole professional lives.

Dr. Thomas S. Kirkbride, the venerable and distinguished Medical Superintendent of the Pennsylvania Hospital for the Insane, writes to your Committee as follows:—

"My opinion of your law can be expressed in a few words. I am quite sure that a jury trial is not required for more than one case out of one hundred that apply for admission, and to compel the other ninety-nine to resort to this process is a great wrong to the afflicted, their friends, and the community. The practical effect must be to consign many to hopeless insanity, for it will prevent proper treatment where alone it is successful in the early stages of the disease. No one will subject a friend to such wanton exposure as long as it can be avoided; and barred rooms at homes, and desolate out-houses will again become the abodes of this afflicted class as they were before the establishment of hospitals. Such a law is a gross interference with the rights of families, and indirectly exposes the community to the risks always attending irresponsible persons having their liberty. It benefits no one, and it seems to me can originate only from a want of knowledge of the subject, and a reliance upon the statements of persons still laboring under mental disease, or actuated by bad motives. These opinions have not been hastily formed, as you know they are the result of more than thirty years' experience among the insane, and the care in that time of considerably over five thousand patients."

Dr. John Curwin, for many years the Medical Superintendent of the State Hospital for the Insane, at Harrisburg, Pennsylvania, writes to the Committee as follows:—

"I believe the system of requiring a jury trial, before a patient can be admitted into a hospital for the insane, to be attended with infinite injury to the insane. It delays treatment; places them in the condition of criminals instead of sick persons; prevents early admission into institutions where alone they can have proper treatment; drags to public gaze the infirm, the weak and defenceless, who ought to be protected, shielded and cared for in the tenderest manner; exposes the private affairs of families and individuals to the gaping curiosity of the idle and busy bodies who delight in scandal; shocks all the finer feelings of all who have any sensibility; compels many to be confined at home, or removed to a great distance, who might be soon cured by prompt and careful treatment, and puts them and their friends to an expense which they should not be called on to meet; and, in fact, I cannot see one redeeming feature in the plan. 'Tis a legislation for exceptional cases, which very rarely arise, and I have sometimes been inclined to suspect the sanity of any one who would insist on such a plan."

Dr. C. H. Nichols, Medical Superintendent of the Government Hospital for the Insane, at Washington, D. C., says, in a discussion of this subject, before the Association of Medical Superintendents of Hospitals for the Insane:—

"If a man suffering from the delirium of fever is bent on jumping out of his window, and a strong shutter or watch is placed in his way, there is just as much reason for raising the senseless cry of 'incarceration,' 'false imprisonment,' 'modern *lettre de cachet*,' 'alarming interference with the liberty of the citizen,' and so on to the end of the catalogue, as in ninety-nine hundred of every thousand persons found under treatment in our institutions for the insane. I speak deliberately. My opportunities for observation are known to you all." * * *

Again the same gentleman says:—

"One of the strongest objections to the judicial process is treating the insane, in one respect, as criminals are treated; that it is subjecting them to a process that has the odor of criminality about it. * * * Regarding insanity as a disease, and the claims of the insane to the tender and skilful treatment they need, as imperative, and not in any manner to be sacrificed, and fully believing that a judicial process to determine the question of insanity is not required in ordinary cases, either by the science or the humanity of the age more than in the delirium of fever, I am very anxious not to take the retrogade step."

Dr. Isaac Ray, our distinguished author on the Medical Jurisprudence of Insanity, says:—

"I have no question, from my own observation, that if you make the law more stringent than it is (that is, by requiring a jury trial), the result will be precisely of that kind that has taken place in England, and will keep out those who ought to be taken care of in our hospitals, and will increase the number of those who will not be placed in hospitals at all. * * *

"We must bear in mind that we are legislating for those who are *really insane*, as well as those who are merely doubtfully so, and consider whether, in order to obviate an evil, that may happen once in a hundred cases, we are to subject the other ninety-nine to an examination that is entirely useless. It is a doubtful kind of legislation that produces more feeling than it prevents. I can easily imagine that in a small country town where everybody knows everybody else's business, an inquisition by a magistrate would scarcely increase the publicity, but it would have a very different effect in a very different kind of community. If that mode should be adopted in our large towns, its needs no Solomon to tell us that we shall have but very few admissions. Insanity is something which people do not care to have made public, and any kind of publicity that subjects the patient to observation and petty curiosity, is objectionable, and any legislation of the kind will operate as prohibitory. The ordinary method of placing patients in a hospital upon medical certificates, which has prevailed from time immemorial, should be legalized, so that we may be able to do that *legally* which we have always done by consent of the community.

Dr. John E. Tyler, Medical Superintendent of the McLean Asylum, at Boston, Massachusetts, says:—"If prompt and early treatment is important for the insane—and we know it is —and if the requisites for admission to a hospital are such extended formalities as to hinder the patient from entering it, of course we are damaging him vitally. But supposing that admission to the hospital is guarded by all the formalities that one can conceive of, I doubt whether the popular distrust would be relieved thereby. The formalities for admission to a hospital should be simple, such as can be complied with without trouble and without publicity. If they are many, and such as will necessarily bring the patient before others, they will certainly keep numbers from the hospital and proper treatment who ought to have it, and thus do great wrong to the insane."

Your Committee believe that these opinions fully accord with those of all other medical men who have given much thought to all that pertains to the welfare of the insane throughout the country.

In conclusion, your Committee submit the following *project of a law*, which is designed more fully to determine certain relations of the insane—to promote the ends of justice, and enforce the claims of an enlightened humanity. This project of a law, it is proper to remark, is the embodiment of the wisdom of the Association of Medical Superintendents of American Institutions for the Insane, unanimously adopted at their annual meeting in June, 1868:—

### AN ACT

*To provide for the admission of certain classes of the Insane into Hospitals for the Insane, and their discharge therefrom.*

SECTION 1. *Be it enacted, etc.*, That insane persons may be placed in a hospital for the insane, by their legal guardians, or by their relatives or friends, in case they have no guardians; but never without the certificate of one or more reputable physicians, after a personal examination, made within one week of the date thereof; and this certificate to be duly acknowledged and sworn to, or affirmed, before some magistrate or judicial officer, who shall certify to the genuineness of the signature, and to the respectability of the signer.

SEC. 2. On a written statement being addressed by some respectable person to any law judge, that a certain person, then confined in a hospital for the insane, is not insane, and is thus unjustly deprived of his liberty, the judge, at his discretion, shall appoint a commission of three persons, one of whom, at least, shall be a physician, and another a lawyer, who shall hear such evidence as may be offered touching the merits of the case, and, without summoning the party to meet them, shall have a personal interview with him, so managed as to prevent him, if possible, from suspecting its objects. They shall report their proceedings to the judge, and if, in their opinion, the party is not insane, the judge shall issue an order for his discharge.

SEC. 3. Whenever any person is acquitted, in a criminal suit, on the ground of insanity, the Jury shall declare this fact in their verdict; and the Court shall order the prisoner to be committed to some place of confinement, for safe keeping, or treatment; there to be retained until he may be discharged in the manner provided in the next section.

Sec. 4. If any law judge shall be satisfied by the evidence presented to him, that the prisoner has recovered, and that the paroxysm of insanity, in which the criminal act was committed, was the first and only one he had ever experienced, he may order his unconditional discharge; if, however, it shall appear that such paroxysm of insanity was preceded by at least one other, then the Court may, in its discretion, appoint a guardian of his person, and to him commit the care of the prisoner, said guardian giving bonds for any damage his ward may commit; *Provided always*, That in case of homicide, or attempted homicide, the prisoner shall not be discharged, unless by the unanimous consent of the Superintendent and the Managers of the Hospital, and the Court before which he was tried.

Sec. 5. Insane persons may be placed in a hospital by order of any law judge, after the following course of proceedings, *viz.:* on statement in writing, of any respectable person, that a certain person is insane, and that the welfare of himself, or of others, requires his restraint, it shall be the duty of the judge to appoint immediately a commission, who shall inquire into, and report upon, the facts of the case. This commission shall be composed of three persons, one of whom, at least, shall be a physician and another a lawyer. In their inquisition, they shall hear such evidence as may be offered touching the merits of the case, as well as the statements of the party complained of, or of his counsel. If, in their opinion, it is a suitable case for confinement, the judge shall issue his warrant for such disposition of the insane person as will secure the object of the measure.

Sec. 6. On statement, in writing, to any law judge, by some friend of the party, that a certain party, placed in a hospital under the fifth section, is losing his bodily health, and that consequently his welfare would be promoted by his discharge; or, that his mental disorder has so far changed its character as to render his further confinement unnecessary, the judge shall make suitable inquisition into the merits of the case, and, according to its result, may, or may not, order the discharge of the party.

Sec. 7. Persons placed in any hospital for the insane may be removed therefrom by parties who have become responsible for the payment of their expenses; *provided*, that such obligation was the result of their own free act and accord, and not of the operation of law, and that its terms require the removal of the patient in order to avoid further responsibility.

Sec. 8. If it shall be made to appear to any law judge that a certain insane person is manifestly suffering from the want of proper care or treatment, he shall order such person to be placed in some hospital for the insane, at the expense of those who are legally bound to maintain them.

Sec. 9. If the Superintendent or officers of any hospital for the insane shall receive any person into the hospital after full compliance with the provisions of the first section of this act, no responsibility shall be incurred by them for any detention in the hospital.

Your Committee beg leave to suggest the appointment of a committee of three members of this Society to memorialize the next Legislature of the State, in favor of enacting the substance of the foregoing project into a law.

